# Flexible Control of Two-Channel Transmission and Group Delay in an Optomechanical System with Double Quantum Dots Driven by External Field

**DOI:** 10.3390/nano11061554

**Published:** 2021-06-12

**Authors:** Faqiang Wang, Weici Liu, Zhongchao Wei, Hongyun Meng, Hongzhan Liu

**Affiliations:** 1 Guangzhou Key Laboratory for Special Fiber Photonic Devices, Laboratory of Nanophotonic Functional Materials and Devices, School of Information and Optoelectronic Science and Engineering, South China Normal University, Guangzhou 510006, China; wzc@scnu.edu.cn (Z.W.); hymeng@scnu.edu.cn (H.M.); lhzscnu@163.com (H.L.); 2 School of Engineering, Guangzhou College of Technology and Business, Foshan 528138, China; liuweici-2002@126.com

**Keywords:** hybrid optomechanical system, electromagnetically induced transparency, group delay, nano-opto-electro-mechanical devices

## Abstract

With the presence of a driving field applied to double quantum dots and a control field applied on the cavity, the transmission performance and group delay effect of a probe field have been theoretically studied in a hybrid optomechanical system (HOMS). Due to the interaction between the mechanical mode and the double quantum dots system, double optomechanically induced transparency (OMIT) arises in the HOMS. With the assistance of a driving field, the system can be tuned to switch on any one of the two OMIT windows, switch on both of the two OMIT windows or switch off both of the two OMIT windows by dynamically adjusting control of the optical field and the driving field. Furthermore, the transmitted probe fields of the two OMIT windows can be tuned to be absorbed or amplified with proper parameters of the driving field and control field. Moreover, the transmission properties of the two OMIT windows are asymmetrical. One can obtain the maximum group delay time of the probe field by optimizing the amplitude and phase of the driving field. These results provide a new way for constructing optically controlled nanostructured photonic switch and storage devices.

## 1. Introduction

Cavity optomechanics has attracted much attention in the past decade [1]. Cavity optomechanics focuses on the study of the interaction between optical and mechanical degrees of freedom, and it builds a connection between the fields of nanophotonics and nanomechanics. The cavity optomechanical system has broad applications in the fields of ground-state cooling of the mechanical mode [2], ultrasensitive sensing [3], optical field squeezing [4], etc. Interestingly, it has been demonstrated that quantum interference leads to optomechanically electromagnetically induced transparency (OMIT) [5,6], which is an analog of electromagnetically induced transparency (EIT) in three-level Λ-type atoms [7]. OMIT has many advanced applications in the optics field, such as slow light [8], charge measurement [9] and light storage [10]. In this paper, we study OMIT in an optomechanical system that consists of an optical cavity that interacts with a mechanical resonator coupled to an artificial two-level system (TLS) realized by double quantum dots (DQDs) driven by an electrical field.

Early OMIT can provide a single transparency window [5] which would limit its application in modern multichannel optical communication. Thus, it is natural to extend single-window OMIT to double-window OMIT and multiple-window OMIT. One method is by coupling the cavity field to two mechanical resonators to realize double-window OMIT in an optomechanical system [11,12,13]; it can be generalized to multiple-window OMIT by introducing more mechanical oscillators. The other important approach is that double-window OMIT can be obtained by introducing the interaction between a mechanical resonator and a two-level system (TLS) [14,15], such as two-level atoms, two-level defects, two-level quantum dot, nitrogen vacancy centers and superconducting qubit circuits. In such an optomechanical system, two transparency windows can be realized because of the destructive interference of the probe field with the Stokes scattering light. Furthermore, one can tune the frequencies of double OMIT by changing the parameters related to the TLS. Double EIT can be used for double-channel optical communication [16], tunable cross-phase modulation [17], high-speed optical switches [18], phonon heat transport controller [19] and a single phonon generator [20,21,22].

Atomic coherence is one of the key points to EIT, while mechanical coherence is the counterpart to OMIT; therefore, mechanical oscillation can be used to control OMIT [23]. Mechanical driving in an optomechanical system would result in mechanical phase-dependent OMIT, where the transmission properties of the probe field are sensitive to the phase of the mechanical mode. The transmission of the probe light field can be controlled by this new degree of freedom [24,25,26,27,28,29,30,31].

Here, we discuss the transmission properties of a probe field incident on a HOMS, where the cavity is pumped by a strong optical control field and the DQD is driven by a weak driving field. We make use of DQDs to construct a pseudo two-level system where its parameters can be changed freely [32]. The single quantum dot can be modeled as a two-level system consisting of the ground state and the single exciton state [33]. The coupling between quantum dot (QD) excitons and acoustic phonons is unavoidable in self-assembled systems. The coupling between exciton and phonon will result in the formation of a polaron [34] and the frequency shift in a QD [35]. Exciton–phonon can also serve as an interface to induce and optically control the quantum state of localized phonon modes [36] or even the macroscopic motion of mechanical oscillators [37]. 

In this paper, we focus on the influences of the driving field applied on DQDs and the properties of the OMIT in an optomechanical system. It is shown that one can not only switch on either of the two OMIT windows but can also switch on both of the two OMIT windows or switch off both of the two OMIT windows by adjusting the driving field to the DQDs. The transmission properties of the two OMIT windows are asymmetrical in our situation. The transmission performances and the group time delay of the output field are discussed in detail.

In this paper, the Hamiltonian model and theoretical formalizations of the HOMS are presented, and the analytical expressions of the probe transmission are obtained based on Langevin equations in Section 2. Then, the effects of the driving field on the transmission performances and the group time delay of the probe light field are presented in Section 3. Finally, Section 4 is the conclusions.

## 2. Model and Theoretical Formalism

**Figure 1:**
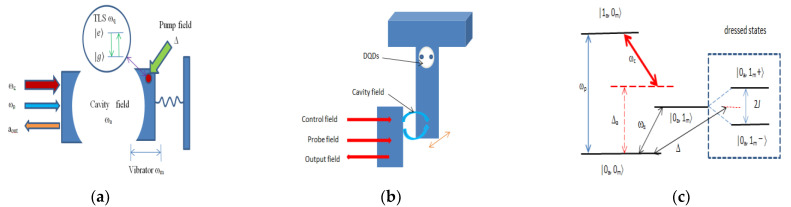
(**a**) Principle diagram of the HOMS. The optical cavity is coupled to a mechanical resonator, which is also coupled to a two-level system (TLS). The cavity is pumped by a control optical field with frequency $\omega_{c}$ and is driven by a weak probe field with frequency $\omega_{p}$, and $a_{out}$ is the output of the cavity field. The TLS is driven by a driving field with frequency $\Delta =\omega_{p}-\omega_{c}$. (**b**) Structure sketch map of the HOMS. The movable mirror can be realized by a cantilever, and the artificial TLS can be realized by double quantum dots (DQDs). (**c**) Energy level diagram of the HOMS with single-photon and single-phonon excitation. $\Delta_{a}=\omega_{a}-\omega_{c}$ $|0_{a},0_{m}\rangle$ and $|1_{a},0_{m}\rangle$ represent the states of the system with 0 or 1 particle, *a* and *m* denote the cavity and mechanical modes, respectively. $|0_{a},1_{m}\pm \rangle$ are the dressed states due to the coupling between the TLS and the mechanical mode. $|0_{a},1_{m}\pm \rangle =\left(\left|0_{a},1_{m},e\rangle \pm \right|0_{a},1_{m},g\right)/\sqrt{2}$.

Figure 1a shows the principle diagram of the HOMS studied in this paper. The optical cavity interacts with a mechanical resonator which is coupled to an artificial TLS driven by an electrical or optical field. The artificial TLS can usually be realized by two-level defects, two-level quantum dots, nitrogen vacancy centers and superconducting qubit circuits [38,39,40]. In this paper, we use DQDs to realize artificial TLSs. The practical structure sketch map of our proposed HOMS is presented in Figure 1b, which has been studied in detail in References [41,42,43]. 

In order to realize the artificial TLS by DQD systems [38,39,44], only one electron can occupy the DQDs at a time because of the small distance between the two QDs. When the left dot is occupied by one electron, it is labeled as  |L〉, and it is labeled as |R〉 if the right dot is occupied by one electron. The state |L〉 corresponds to the ground state and |R〉 corresponds to the excited state [45]. The energy level and the tunneling amplitude of DQDs can be tuned by adjusting the gate voltages [38]. The interaction between a single QD and the nanomechanical mode can be described by the spin-boson model because the longitudinal strain will affect the energy of the QD exciton states due to deformation potential coupling [46,47], which has been studied experimentally [37]. If the energy level difference between the two QDs is much less than the tunneling amplitude between the two QDs, the interaction between the DQDs and the mechanical mode can be simplified to the following Hamiltonian [48,49,50], $H_{qm}=\hbar J\left(\sigma_{+}b+\sigma_{-}b^{†}\right)$, where rotating wave approximation has been made. Here, *J* is the coupling coefficient, and $\sigma_{+}$($\sigma_{-})$ is the raising (lowering) operator of the TLS. $\sigma_{+}=|e\rangle \langle g|$ and $\sigma_{-}=|g\rangle \langle e|$ are the Pauli operators of the TLS, with |g〉 representing the ground hybridized eigenstate and |e〉 representing the excited hybridized eigenstate of the isolated DQDs [48]. The vibration energy density becomes larger as the effective phonon mode volume decreases. Large vibrations will modify the energy of the electronic states of quantum dots through deformation potential coupling [46]. *J* will increase with the decrease of effective phonon mode volume.

$H_{om}=-\hbar g_{0}a^{†}a\left(b^{†}+b\right)$ is the Hamiltonian between the cavity and the mechanical mode. Here, $a^{†}\left(a\right)$ is the creation (annihilation) operator of the cavity mode, and $b^{†}\left(b\right)$ is the creation (annihilation) operator of the mechanical mode. $g_{0}$ is the vacuum optomechanical coupling strength between the cavity field and the mechanical mode, which is expressed in frequency.

A weak probe field with frequency $\omega_{p}$ and a strong control field with frequency $\omega_{c}$ are used to drive the cavity field. Furthermore, applying an alternative driving voltage with frequency $\Delta =\omega_{p}-\omega_{c}$ and phase $\phi_{q}$ to the plunger gates of DQDs to induce a small amplitude oscillation of energy level difference between the two QDs, where the Hamiltonian of the driving effect is $\hbar \varepsilon^{\prime }\sin\left(\Delta t+\phi_{q}\right)\left\{|L\rangle \langle L|-|R\rangle \langle R|\right\}$ [48,51], and ε’ is proportional to the amplitude of driving voltage. If the energy level difference between the two QDs is much less than the tunneling amplitude between the two QDs, rewriting the driving Hamiltonian in the space of hybridized eigenstates of the isolated DQDs [48] and making rotating wave approximation, then in a rotating frame with the frequency of $\omega_{c}$, the total Hamiltonian could be rewritten as:

(1) $$H=\hbar \Delta_{a}a^{†}a+\hbar \omega_{m}b^{†}b+\hbar \omega_{q}\sigma_{z}/2+H_{om}+H_{qm}+H_{dr}$$

(2) $$H_{dr}=i\hbar \varepsilon_{c}\left(a^{†}-a\right)+i\hbar \varepsilon_{p}\left(a^{†}e^{-i\Delta t-i\phi_{pc}}-ae^{i\Delta t+i\phi_{pc}}\right)+i\hbar \varepsilon_{q}\left(\sigma_{+}e^{-i\Delta t-i\phi_{q}}-\sigma_{-}e^{i\Delta t+i\phi_{q}}\right)$$

where $\Delta_{a}=\omega_{a}-\omega_{c}$ is the detuning between the cavity-field frequency $\omega_{a}$ and the control-field frequency $\omega_{c}$, the mechanical mode frequency is $\omega_{m}$, and $\omega_{q}$ is the transition frequency of the TLS. Here, $\sigma_{z}=|e\rangle \langle e|-|g\rangle \langle g|$, and $\phi_{pc}=\phi_{p}-\phi_{c}$ is the phase difference between the control optical field and the probe field. $\varepsilon_{c}\;$ is the amplitude of the control field, $\varepsilon_{p}$ is the amplitude of the probe field, and $\varepsilon_{q}=\varepsilon^{\prime }/2$ is the driving amplitude of the TLS induced by the driving field in the space of hybridized eigenstates of the isolated DQDs [48].

From the total Hamiltonian of the HOMS, one can get the quantum Langevin equations (QLEs) of the system as follows:

(3a) $$\dot{a}=-\left(\gamma_{a}+{i\Delta }_{a}\right)a+ig_{0}a\left(b^{†}+b\right)+\varepsilon_{c}+\varepsilon_{p}e^{-i\Delta t-i\phi_{pc}}$$

(3b) $$\dot{b}=-\left(\gamma_{m}+i\omega_{m}\right)b+ig_{0}a^{†}a-iJ\sigma_{-}$$

(3c) $${\dot{\sigma }}_{-}=-\left(\gamma_{q}/2+i\omega_{q}\right)\sigma_{-}+iJ\sigma_{z}b+\varepsilon_{c}-\varepsilon_{q}\sigma_{z}e^{-i\Delta t-i\phi_{q}}$$

(3d) $${\dot{\sigma }}_{z}=-\gamma_{q}\left(\sigma_{z}+1\right)-2iJ\left(\sigma_{+}b-b^{†}\sigma_{-}\right)+\varepsilon_{c}+2\varepsilon_{q}\left(\sigma_{+}e^{-i\Delta t-i\phi_{q}}+\sigma_{-}e^{i\Delta t+i\phi_{q}}\right)$$

where $\gamma_{a}$, is the decay rate of the cavity field, $\gamma_{m}$ is the decay rate of mechanical resonator, and $\gamma_{q}$ is the decay rate of the TLS. In this paper, we neglect the quantum and thermal noise because we focus on the mean optical response of the HOMS. Usually, the performance of the system could not be deteriorated by the thermal noise terms. However, the quantum and thermal noise are more critical in deteriorating the performance of the single-photon device based on the system proposed here. To reduce the detrimental effects of thermal noise, one can cool the system down to cryogenic temperatures. After some algebra, one can get the systems steady-state solutions as the following:

(4a) $$\alpha ={\langle a\rangle }_{s}=\varepsilon_{c}/\left(\gamma_{a}+i\Delta_{a}^{’}\right)$$

(4b) $$\beta ={\langle b\rangle }_{s}=\left(ig_{0}{\left|\alpha \right|}^{2}-iJL_{0}\right)/\left(\gamma_{m}+i\omega_{m}\right)$$

(4c) $$L_{0}={\langle \sigma \rangle }_{-}{}_{s}=iJ\beta W_{0}/\left(\gamma_{q}/2+i\omega_{q}\right)$$

(4d) $$W_{0}=\left(\gamma_{q}^{2}+4\omega_{q}^{2}\right)/\left(\gamma_{q}^{2}+4\omega_{q}^{2}+8J^{2}{\left|\beta \right|}^{2}\right)$$

where $\Delta_{a}^{’}=\Delta_{a}-g\left(\beta^{*}+\beta \right)$. The steady-state solutions are the same as that of the HOMS without a driving field applied on the TLS [15].

Here, the cavity is assumed to be driven at the red sideband regime (i.e., $\Delta_{a}\approx \omega_{m}$). Under the condition of the resolved sideband limit $\omega_{m}\gg \gamma_{a},\;g\alpha$, we can make use of rotating wave approximation (RWA) here [28]. Thus, we can set $a=\alpha +\delta ae^{-i\Delta t}$, $b=\beta +\delta be^{-i\Delta t}$*,* $\sigma_{-}=L_{0}+\delta \sigma_{-}e^{-i\Delta t}$*,* $\sigma_{z}=W_{0}+\delta \sigma_{z}e^{-i\Delta t}$, then the following equations are obtained:

(5a) $$\delta \dot{a}=-\left(\gamma_{a}+i\Delta_{a}-i\Delta \right)\delta a+iG\delta b+\varepsilon_{p}e^{-i\phi_{pc}}$$

(5b) $$\dot{\delta b}=-\left(\gamma_{m}+i\omega_{m}-i\Delta \right)\delta b+iG^{*}\delta a-iJ\delta \sigma_{-}$$

(5c) $$\delta {\dot{\sigma }}_{-}=-\left(\gamma_{q}/2+i\omega_{q}-i\Delta \right)\sigma_{-}+iJ(\beta \delta \sigma_{z}+W_{0}\delta b)-\varepsilon_{q}W_{0}e^{-i\phi_{q}}$$

(5d) $$\delta {\dot{\sigma }}_{z}=-\left(\gamma_{q}-i\Delta \right)\delta \sigma_{z}-2iJ\left(L_{0}^{*}\delta b-\beta^{*}\delta \sigma_{-}\right)+2\varepsilon_{q}L_{0}^{*}e^{-i\phi_{q}}$$

where $G=g_{0}\alpha$. Under the steady-state condition, one can obtain

(6) $$\langle \delta a\rangle =\frac{\xi \varepsilon_{p}e^{-i\phi_{pc}}+\left\{2iJ^{2}G\beta L_{0}^{*}-GJW_{0}\left(\gamma_{q}-i\Delta \right)\right\}\varepsilon_{q}e^{-i\phi_{q}}}{\xi \left(\gamma_{a}+i\omega_{1}\right)+\psi {\left|G\right|}^{2}}$$

here, $\xi =\psi \left(\gamma_{m}+i\omega_{2}\right)+2iJ^{3}\beta L_{0}^{*}-J^{2}W_{0}\left(\gamma_{q}-i\Delta \right)$*,* $\psi =\left(\gamma_{q}/2+i\omega_{3}\right)\left(\gamma_{q}-i\Delta \right)+2J^{2}{\left|\beta \right|}^{2}$, $\omega_{1}=\Delta_{a}^{’}-\Delta$, $\omega_{2}=w_{m}-\Delta$, $\omega_{3}=\omega_{q}-\Delta$. The cavity field oscillates in the frequency of Δ. It reflects the Rabi oscillations of the dressed states.

Using the input–output theory, we could obtain $\langle \varepsilon_{\mathrm{out}}\rangle +\varepsilon_{c}+\varepsilon_{p}e^{-i\Delta t-i\phi_{\mathrm{pc}}}=2{\eta \gamma }_{a}\langle \delta a\rangle$ [5,28]. Here, η is the coupling efficiency [52]. The quadratures of the output field at the frequency of the probe field are defined as $\;\varepsilon_{T}=\frac{2\eta \gamma_{a}\langle \delta a\rangle }{\varepsilon_{p}e^{-i\phi_{pc}}}=\upsilon_{p}+i{\tilde{\upsilon }}_{p}$ [5]. Thus, the transmission coefficient of the probe field can be obtained as [5,28]:

(7) $$t_{p}=\frac{k_{e}\langle \delta a\rangle -\varepsilon_{p}e^{-i\phi_{pc}}}{\varepsilon_{p}e^{-i\phi_{pc}}}=t_{1}+t_{2}$$

with

(8) $$t_{1}=\frac{\xi k_{e}}{\xi \left(\gamma_{a}+i\omega_{1}\right)+\psi {\left|G\right|}^{2}}-1$$

(9) $$t_{2}=\frac{iGk_{e}\left\{2J^{2}\beta L_{0}^{*}+iJW_{0}\left(\gamma_{q}-i\Delta \right)\right\}re^{-i\phi }}{\xi \left(\gamma_{a}+i\omega_{1}\right)+\psi {\left|G\right|}^{2}}$$

where $r=\varepsilon_{q}/\varepsilon_{p}$, $\phi =\phi_{q}-\phi_{pc}$ which is the phase difference between the driving field and $\phi_{pc}$, and $k_{e}=2\eta \gamma_{a}$ is the external decay rate of the cavity [52]. Here, $t_{1}$ is the corresponding part influenced by the control optical field, and $t_{2}$ is the corresponding part influenced by the driving field on the probe transmission. The probe transmission coefficient is dominated by the interference between $t_{1}$ and $t_{2}$, especially if it is remarkably affected by the phase difference ϕ.

## 3. Results and Discussion

In order to study the phase-controlled probe transmission spectrum, we numerically calculate the probe transmission coefficient with the parameters chosen from previous works [14,15,28]. We assume: $\omega_{m}=\omega_{q}=2\pi \times 100\;\mathrm{MHz}$, $g_{0}=2\pi \times 10\;\mathrm{MHz}$, $\gamma_{a}=2\pi \times 3\;\mathrm{MHz}$, $\gamma_{m}=2\pi \times 2\;\mathrm{kHz}$,$\;\gamma_{q}=2\pi \times 0.1\;\mathrm{MHz}$, J=2π×1 MHz, $\varepsilon_{c}=2\pi \times 10\;\mathrm{MHz}$, η=0.45, and $\Delta_{a}=\omega_{m}$.

### 3.1. Phase-Controlled Two-Channel Selective Transmission

**Figure 2:**
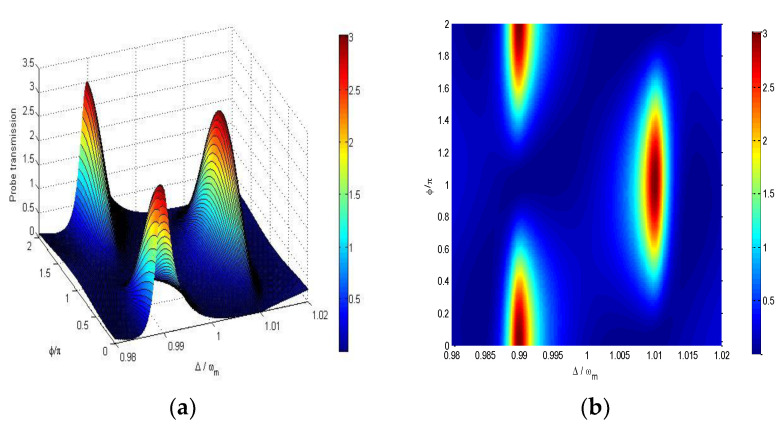
(**a**) A 3D mesh picture and (**b**) contour image of the probe transmission ${\left|t_{p}\right|}^{2}$ versus ϕ/π and probe-control field detuning $\Delta ⁄\omega_{m}$, $\;\omega_{m}=\omega_{q}=2\pi \times 100\;\mathrm{MHz}$, $\;g_{0}=2\pi \times 10\;\mathrm{MHz}$, $\;\gamma_{q}=2\pi \times 3.2\;\mathrm{MHz}$, $\gamma_{m}=2\pi \times 2\;\mathrm{KHz}$, $\gamma_{q}=2\pi \times 0.1\;\mathrm{MHz}$, J=2π×1 MHz, $\varepsilon_{c}=2\pi \times 10\;\mathrm{MHz}$, *r* = 0.35, *η* = 0.45 and Δ_*a*_ = *ω*_*m*_.

Figure 2 shows that the probe transmission ${\left|t_{p}\right|}^{2}$ varies with ϕ/π and the detuning between probe field and pump field $\Delta ⁄\omega_{m}$. It shows that the variations of the probe transmission at $\Delta =\omega_{m}\pm J$ with the phase difference $\phi =\phi_{q}-\phi_{pc}$ are asymmetric. The probe transmission ${\left|t_{p}\right|}^{2}$ attains the maximum value at $\Delta /\omega_{m}=0.99$ while it reaches the minimum value at $\Delta /\omega_{m}=1.01$ for fixed ϕ=0, and vice versa for ϕ=π. We can select which channel of $\omega_{m}\pm J$ to switch on by varying the phase of the driving field applied on DQDs at the fixed $\phi_{pc}=\phi_{p}-\phi_{c}$. 

It is also found that the probe transmission $|t_{p}{|}^{2}$ can be larger than unity in the two channels $\Delta =\omega_{m}\pm J$ when ϕ varies, which means that the output probe field can be amplified in certain variation ranges of ϕ. The probe field can be amplified because of the following transition process that the system transitions from the ground state $|0_{a},0_{m}\rangle$ to $|1_{a},0_{m}\rangle$ through absorbing one driving field particle and one pump field particle and then emitting one probe field particle with the transition from $|1_{a},0_{m}\rangle$ down to $|0_{a},0_{m}\rangle$ (see Figure 1c), in which the energy of pump field and driving field can be transferred to the probe field. 

**Figure 3:**
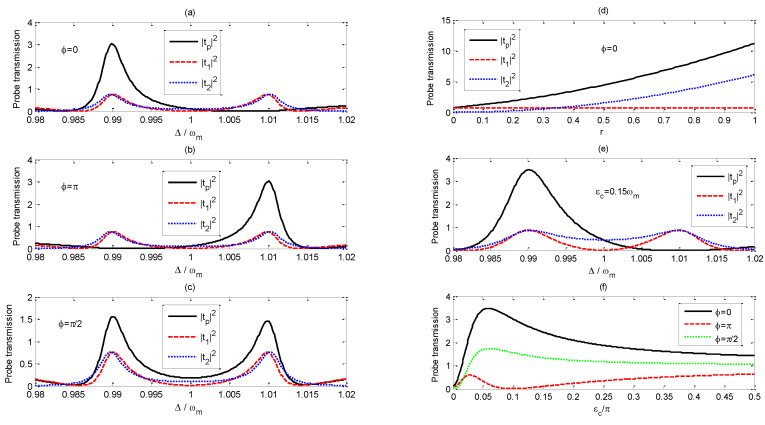
Curves of ${\left|t_{p}\right|}^{2}$, ${\left|t_{1}\right|}^{2}$, ${\left|t_{2}\right|}^{2}$ vary with the probe-pump field detuning $\Delta /\omega_{m}$ when ϕ equals to (**a**) 0, (**b**) π and (**c**) π/2, respectively. (**d**) Curves of ${\left|t_{p}\right|}^{2}$, ${\left|t_{1}\right|}^{2}$, ${\left|t_{2}\right|}^{2}$ at $\Delta =\omega_{m}-J$ vary with $r=\mu \varepsilon_{q}/\varepsilon_{p}$ when the phase difference ϕ=0. (**e**) Curves of ${\left|t_{p}\right|}^{2}$, ${\left|t_{1}\right|}^{2}$, ${\left|t_{2}\right|}^{2}$ vary with the probe-pump field detuning $\Delta /\omega_{m}$ for ϕ=0 when the pump field amplitude $\varepsilon_{c}$ equals to $0.15\omega_{m}$ and r=0.52. (**f**) Curves of ${\left|t_{p}\right|}^{2}$ at $\Delta =\omega_{m}-J$ vary with pump field amplitude $\varepsilon_{c}/2\pi$ for different phase difference ϕ when *r*=0.35. The other parameters are the same as those in Figure 2.

Figure 3a–c exhibit that the curves of ${\left|t_{p}\right|}^{2}$, ${\left|t_{1}\right|}^{2}$, ${\left|t_{2}\right|}^{2}$ vary with the probe-pump field detuning $\Delta /\omega_{m}$ when ϕ is set to (a) 0, (b) π and (c) π/2, respectively. ${\left|t_{1}\right|}^{2}$ represents the probe transmission without the driving field applied on the DQDs system, and double OMIT is observed around $\Delta =\omega_{m}\pm J$ due to the interference of the probe field with the anti-Stokes field resulting from the radiation pressure [14,15,28]. There are two main transition pathways as the system transitions from the ground state $|0_{a},0_{m}\rangle$ to $|1_{a},0_{m}\rangle$, which are $\left|0_{a},0_{m}\rangle \to \right|0_{a},1_{m}\pm \rangle \to |1_{a},0_{m}\rangle$ and $\left|0_{a},0_{m}\rangle \to \right|1_{a},0_{m}\rangle$. The destructive interference between two excitation pathways leads to the presence of double OMITs [14]. ${\left|t_{2}\right|}^{2}$ represents the influence of the driving field applied on the DQDs on the probe transmission. The driving field can affect the phase and probability amplitude of the transition pathway of the transition process of $\left|0_{a},0_{m}\rangle \to \right|0_{a},1_{m}\pm \rangle \to |1_{a},0_{m}\rangle$, which results in the controlling of the transmission properties of the probe field by adjusting the amplitude and phase of the driving field.

Comparing the red dashed line with the blue dotted line in Figure 3a,b, we find ${\left|t_{1}\right|}^{2}$ and ${\left|t_{2}\right|}^{2}$ remain the same as ϕ varies. However, if we set  ϕ=0, ${\left|t_{p}\right|}^{2}$ is much larger than both ${\left|t_{1}\right|}^{2}$ and ${\left|t_{2}\right|}^{2}$ around $\Delta =\omega_{m}-J$*(*$\Delta /\omega_{m}=0.99$), which results from the constructive interference between $t_{1}$ and $\;t_{2}$, while ${\left|t_{p}\right|}^{2}$ is almost zero around $\Delta =\omega_{m}+J$ *(*$\Delta /\omega_{m}=1.01$) because of the destructive interference between $t_{1}$ and $\;t_{2}$. Figure 3d demonstrates that ${\left|t_{1}\right|}^{2}$ does not vary with an increase of the amplitude of the driving field, but ${\left|t_{2}\right|}^{2}$ increases monotonically as *r* varies. With the increase of the driving-field amplitude, ${\left|t_{p}\right|}^{2}$ at $\Delta /\omega_{m}=0.99$ becomes larger and larger due to the constructive interference between $t_{1}$ and $\;t_{2}$. Figure 3b demonstrates that the transmission properties of the probe field ${\left|t_{p}\right|}^{2}$ around $\Delta /\omega_{m}=0.99$ and $\Delta /\omega_{m}=1.01$ as ϕ=π is adverse to that for the situation as ϕ=0.

From Figure 3c, one can find that double OMIT is observed around $\Delta =\omega_{m}\pm J$ when ϕ=π/2, although the maximum probe transmissions around $\Delta =\omega_{m}\pm J$ are little smaller than the corresponding maximum values on Figure 3a,b. Tuning the phase difference ϕ form π/2 to π, the probe transmission around $\Delta /\omega_{m}=0.99$ would be tuned from remarkable amplification to perfect absorption, while the variation of the probe transmission around $\Delta /\omega_{m}=1.01$ goes in the opposite direction.

In order to obtain the enhanced amplitude of the probe transmission coefficient ${\left|t_{p}\right|}^{2}$ at a selected channel, such as $\Delta =\omega_{m}-J$ ($\Delta /\omega_{m}=0.99$), one could increase parameter *r*. However, the probe transmission coefficient ${\left|t_{p}\right|}^{2}$ at the other channel $\Delta /\omega_{m}=1.01$ will no longer stay at zero if *r* varies from the original value of 0.35. If one wants to switch on one channel and switch off the other at the same time, one should adjust the other parameters; for example, one can set the pump field amplitude $\varepsilon_{c}=0.15\omega_{m}$ as shown in Figure 3e. 

Figure 3f shows that the probe transmissions ${\left|t_{p}\right|}^{2}$ at $\Delta /\omega_{m}=0.99$ vary with the increase of the pump-field amplitude $\varepsilon_{c}/2\pi$ for different phase difference ϕ. For ϕ=0, the probe transmission ${\left|t_{p}\right|}^{2}$ at $\Delta /\omega_{m}=0.99$ would be enhanced by the constructive interference between $t_{1}$ and $\;t_{2}$, but it is suppressed by the destructive interference for ϕ=π. At the crossover point, the complete destructive interference between $t_{1}$ and $\;t_{2}$ results in ${\left|t_{p}\right|}^{2}=0$ for ϕ=π. Thus, one can control the amplitude of ${\left|t_{p}\right|}^{2}$ by varying the pump-field amplitude $\varepsilon_{c}$.

**Figure 4:**
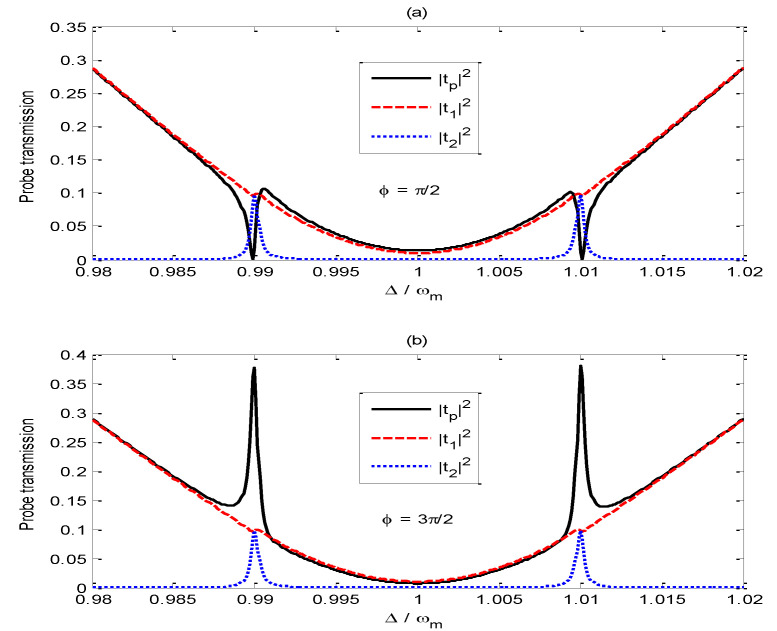
Curves of ${\left|t_{p}\right|}^{2}$, ${\left|t_{1}\right|}^{2}$, ${\left|t_{2}\right|}^{2}$ vary with the probe-pump field detuning $\Delta /\omega_{m}$ for (**a**) ϕ=π/2 and (**b**) ϕ=3π/2. The other parameters are the same as those in Figure 2 except for $\varepsilon_{c}=0.0055\omega_{m}$

Figure 4 shows how the curves of ${\left|t_{p}\right|}^{2}$, ${\left|t_{1}\right|}^{2}$, ${\left|t_{2}\right|}^{2}$ vary with the probe-pump field detuning $\Delta /\omega_{m}$ as the phase difference ϕ is set to (a) π/2 and (b) 3π/2, respectively. From the red dashed line in Figure 4a, one can find that ${\left|t_{1}\right|}^{2}$ around the two OMIT windows is very small because the small pump-field amplitude reduces the interference between the probe field and the anti-Stokes field. From the blue dotted line in Figure 4a, we can find that ${\left|t_{2}\right|}^{2}$ is obviously enhanced around the two OMIT windows, and ${\left|t_{2}\right|}^{2}$ has the same value as that of ${\left|t_{1}\right|}^{2}$ at $\Delta =\omega_{m}\pm J$. Comparing Figure 4a with Figure 4b, we can find that the deconstructive interference between $t_{1}$ and $\;t_{2}$ leads to ${\left|t_{p}\right|}^{2}=0$ at $\Delta =\omega_{m}\pm J$ for ϕ=π/2, while the constructive interference between $t_{1}$ and $\;t_{2}$ results in the maximum of the probe transmission ${\left|t_{p}\right|}^{2}$ at $\Delta =\omega_{m}\pm J$ for ϕ=3π/2.

### 3.2. Group Time Delay Effect

Since rapid phase dispersion would result in a group time delay effect within the transparency window, we will discuss how to control the phase dispersion by tuning the pump field and the driving field in this subsubsection.

The group time delay of the transmitted probe field is defined as [28]:

(10) $$\tau_{g}=\frac{d\phi_{t}\left(\omega_{p}\right)}{d\omega_{p}}=\frac{d\left\{arg\left[t_{p}\left(\omega_{p}\right)\right]\right\}}{d\omega_{p}}$$

**Figure 5:**
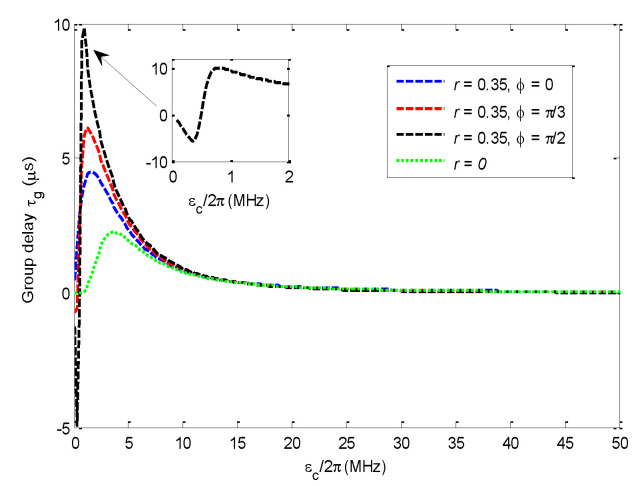
Curves of group time delay $\tau_{g}$ vary with the pump-field amplitude $\varepsilon_{c}/2\pi$ and driving field amplitude at $\Delta /\omega_{m}=0.99$ for different phase differences. The other parameters are the same as those in Figure 2.

Figure 5 gives the curves of the group time delay $\tau_{g}$ that vary with the pump-field amplitude $\varepsilon_{c}/2\pi$ for different driving-field amplitudes. If the driving field amplitude is set to zero, the maximum group delay time is $\tau_{g}=2.24\mu s$. The group time delay $\tau_{g}$ is obviously influenced by the additional driving field, especially if it is dominated by the phase difference ϕ. 

If *r* = 0.35 and ϕ=0, the maximum group delay time $\tau_{g}$ at $\Delta /\omega_{m}=0.99$ can be extended to about 4.47μs with the amplified probe field; If we set *r* = 0.35 and ϕ=π/3, the maximum group delay $\tau_{g}$ at $\Delta /\omega_{m}=0.99$ increases to about 6.12μs; 

The maximum group time delay $\tau_{g}$ at $\omega_{m}\pm J$ can be extended to about 9.82μs at $\varepsilon_{c}/2\pi =0.9\;\mathrm{MHz}$ if *r* = 0.35 and ϕ=π/2 for dual-channel operation mode. Furthermore, the fast light effect appears because the group time delay $\tau_{g}$ is negative around $\varepsilon_{c}/2\pi =0.3\;\mathrm{MHz}$. From Figure 3f, we can find that the probe transmission coefficient at $\varepsilon_{c}/2\pi =0.9\;\mathrm{MHz}$ is about 0.1, and the probe transmission coefficient at $\varepsilon_{c}/2\pi =0.3\;\mathrm{MHz}$ is about 0.03, while the probe transmission around the negative group delay region is approximately equal to zero in reference [28]. Thus, one should make a trade-off between long group time delay and high probe transmittance. Parameters should be selected to guarantee that ${\left|t_{p}\right|}^{2}$ is non-zero.

**Figure 6:**
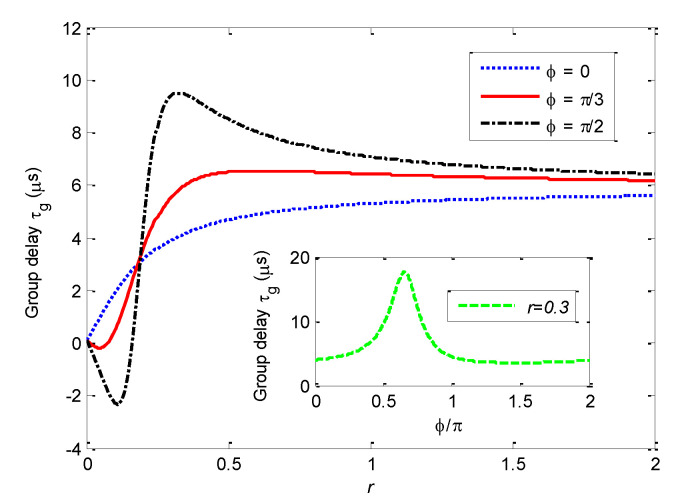
Curves of group delay $\tau_{g}$ at $\Delta /\omega_{m}=0.99$ vary with $r=\mu \varepsilon_{q}/\varepsilon_{p}$ for different phase difference. The inset of Figure 6 shows the curve of $\tau_{g}$ varies with *ϕ* at *r* = 0.3. The other parameters are the same as those in Figure 2 except for $\varepsilon_{c}/2\pi =0.9\;\mathrm{MHz}$.

For a fixed pump field, the group time delay effect can be affected by the amplitude and the phase of the driving field. Figure 6 shows that $\tau_{g}$ varies with *r* for ϕ=0, π/3, and π/2, respectively. One can find that $\tau_{g}$ at $\Delta =\omega_{m}-J$ *(*$\Delta /\omega_{m}=0.99$*)* raises monotonically as *r* increases at ϕ=0. For ϕ=π/3 and π/2, the group delay firstly grows and then descends gradually as *r* increases. The group delay time can be extended as the phase difference ϕ increases.

In addition, the inset of Figure 6 shows that the group delay $\tau_{g}$ varies with ϕ for fixed *r* = 0.3. It can be found that the group delay time $\tau_{g}$ has a maximum as the variation of ϕ for fixed *r*. 

**Figure 7:**
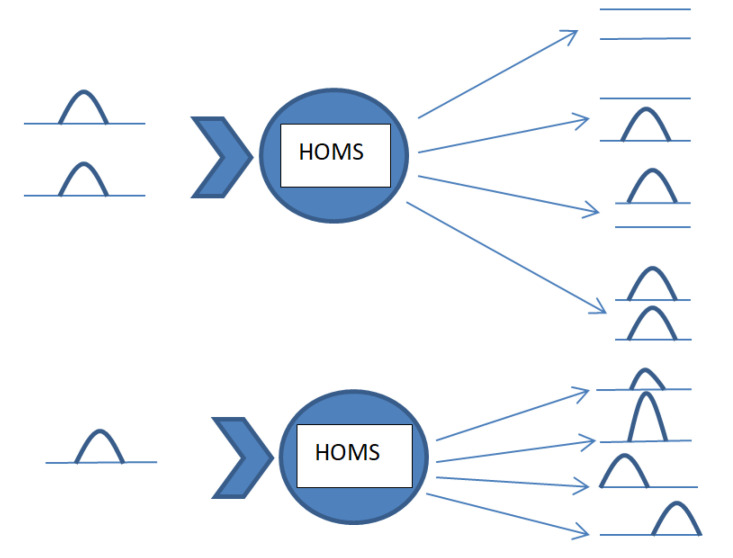
A schematic figure of the possible functions that can be realized by the proposed system.

Figure 7 presents a schematic figure of the possible functions that can be realized by the system proposed here. The system can realize a two-channel selective-switch function by adjusting the phase of the driving field applied on the DQDs, and one can turn on any single channel, turn on both channels or turn off both channels by modulating the driving-field phase. The transmitted probe field would be tuned from remarkable amplification to perfect absorption by changing the driving-field phase. Furthermore, the group time delay can reach a maximum, and negative group delay can be realized with nonzero probe field transmission for proper parameters.

The properties of the transmitted probe field can be controlled flexibly by adjusting the driving field and the pump optical field. 

## 4. Conclusions

In summary, the driving field can obviously affect the transmission properties of the weak probe field through the HOMS.

Firstly, the system can be tuned to switch on any one channel of the two OMIT windows, switch on both channels of the two OMIT windows or switch off both channels of the two OMIT windows by modulating the driving-field amplitude and phase. The transmission properties of two OMIT windows are asymmetrical.

Secondly, the transmission of the probe field in the double OMIT window can be tuned into amplification or absorption, and the group time delay of the probe field can be obviously enhanced if one chooses proper parameters for amplitude and phase for the driving field on DQDs. 

In brief, the system can act as a two-channel selective switch and a light storage, and its properties can be adjusted by dynamically modulating the driving field and the pump optical field.

## Data Availability

The authors confirm that the data supporting the findings of this study are available within the article. All data supporting the findings of this study are available from the corresponding author [F.W.] on request.
